# Structural Synaptic Plasticity Has High Memory Capacity and Can Explain Graded Amnesia, Catastrophic Forgetting, and the Spacing Effect

**DOI:** 10.1371/journal.pone.0096485

**Published:** 2014-05-23

**Authors:** Andreas Knoblauch, Edgar Körner, Ursula Körner, Friedrich T. Sommer

**Affiliations:** 1 Engineering Faculty, Albstadt-Sigmaringen University, Albstadt, Germany; 2 Honda Research Institute Europe, Offenbach am Main, Germany; 3 Redwood Center for Theoretical Neuroscience, University of California, Berkeley, California, United States of America; University of Sheffield, United Kingdom

## Abstract

Although already William James and, more explicitly, Donald Hebb's theory of cell assemblies have suggested that activity-dependent rewiring of neuronal networks is the substrate of learning and memory, over the last six decades most theoretical work on memory has focused on plasticity of existing synapses in prewired networks. Research in the last decade has emphasized that structural modification of synaptic connectivity is common in the adult brain and tightly correlated with learning and memory. Here we present a parsimonious computational model for learning by structural plasticity. The basic modeling units are “potential synapses” defined as locations in the network where synapses can potentially grow to connect two neurons. This model generalizes well-known previous models for associative learning based on weight plasticity. Therefore, existing theory can be applied to analyze how many memories and how much information structural plasticity can store in a synapse. Surprisingly, we find that structural plasticity largely outperforms weight plasticity and can achieve a much higher storage capacity per synapse. The effect of structural plasticity on the structure of sparsely connected networks is quite intuitive: Structural plasticity increases the “effectual network connectivity”, that is, the network wiring that specifically supports storage and recall of the memories. Further, this model of structural plasticity produces gradients of effectual connectivity in the course of learning, thereby explaining various cognitive phenomena including graded amnesia, catastrophic forgetting, and the spacing effect.

## Introduction

Traditionally, learning and memory are attributed to *weight plasticity*, that is, the modification of the strength of existing synapses according to variants of the Hebb rule [1]–[5]. Although the theory of weight plasticity has been crucially important in neuroscience and applications of artificial neural networks, it could not easily explain various fundamental memory-related effects in cognitive psychology such as graded amnesia, the prevention of catastrophic forgetting, and the spacing effect.

Another form of synaptic plasticity is *structural plasticity*, that is, the creation and erasure of synapses [6]–[13]. Originally thought of setting up connectivity during development [14]–[16] or after injuries [17], [18], it has recently been shown to correlate with memory formation and learning in the healthy adult brain [19]–[23].

Here we introduce and analyze a simple computational model of structural plasticity which exhibits surprisingly high memory capacity and is able to explain the mentioned cognitive effects. A key to understanding the role of structural plasticity in memory has to do with the observation that the brain, even its most densely connected local circuits, is far from being fully connected [24], [25]. Thus, for any given network computation, the existing synapses may or may not provide the optimal structure of the network. To assess the match between existing synapses and the synapses required by a computation, we define *effectual connectivity* as the fraction of required synapses that are present in the network. By erasure and creation of synapses, structural plasticity can “migrate” synapses and thereby increase the effectual connectivity for a given network function. By integrating our model with well-known Hopfield- or Willshaw-type neural network models of memory storage and retrieval [16], [26], [27] we can quantitatively asses the benefits of structural plasticity compared to weight plasticity. In section 0.6 we show that ongoing structural plasticity can strongly increase storage capacity for sparsely connected networks, which is in line with related approaches counting possible synaptic network configurations [28]–[30] or analyzing storage capacity for structural plasticity during development [15], [16]. Moreover, our theory of structural plasticity suggests immediate explanations for various memory phenomena [31]–[33]. In particular, in section 7 we analyze the role of structural synaptic plasticity in cortico-hippocampal memory replay and consolidation [34], [35], preventing catastrophic forgetting in brains [36], [37], graded retrograde amnesia following brain lesions [38]–[40], and the pedagogically relevant spacing effect of learning [41]–[43].

## Concepts and Models

### 1 Synapse Ensembles and Effectual Connectivity

**Figure 1:**
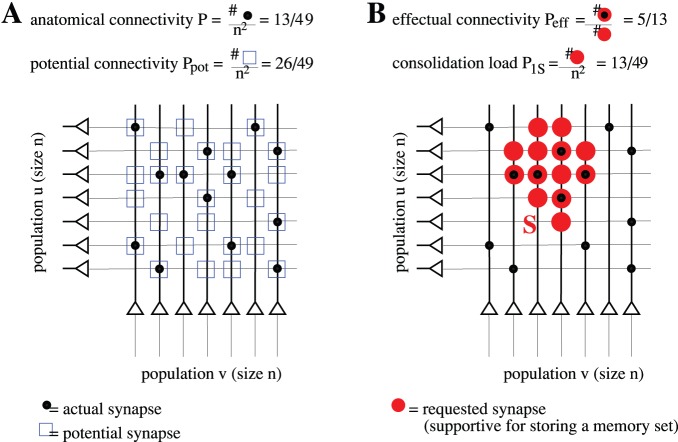
Definitions of network connectivity. Illustration of different connectivity measures for a synaptic network 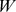 connecting neuron populations 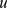 to 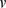 (which may be identical for recurrent networks). **A,** *Anatomical connectivity* 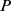 and *potential connectivity* 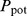 are fractions of neuron pairs 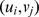 connected by an actual (black circles) and potential synapse (blue rectangles), respectively. **B,** The *consolidation signal* 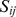 specifies the ensemble of neuron pairs that request a synapse (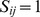, red circles) to support storage of a given memory set. The corresponding *effectual connectivity* 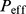 is then the fraction of neuron pairs requesting a synapse that are already connected by an actual synapse. The *consolidation load* 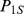 is the fraction of neuron pairs that request a synapse.

Common memory theories based on neural associative network models consider only Hebbian-type weight plasticity in networks with fixed structure, thus, neglecting processes involving structural plasticity. Such models predict that the maximal information that can be stored in a given neural network increases in proportion to the number of synaptic connections rather than number of neurons. Therefore, *storage capacity* 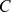 is often expressed in terms of stored information per synapse. For example, 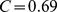 bit per synapse (bps) for networks of binary synapses [26], [44], or 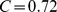 bps for real-valued synaptic weights [45], [46]. To judge how many memories can be stored in a network 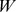 connecting two neuron populations 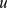 and 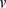 each comprising 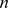 neurons, it is therefore important to know the *anatomical network connectivity*

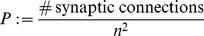
(1)


defined as the chance that there is a synaptic connection between two randomly chosen neurons (Fig. 1A).

For memory theories including structural plasticity the situation is different because we can assume that processes including generation of new synapses, consolidation of useful synapses, elimination of useless synapses, and maintenance of anatomical connectivity at a given level 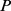 will effectively “migrate” synapses to locations that are most appropriate for storing a particular set of memories. Evidently, anatomical connectivity will then be a bad predictor of storage capacity. Rather storage capacity will depend crucially on the number of locations where a synapse could potentially be generated. Such locations have been called potential synapses [29], where *potential network connectivity*

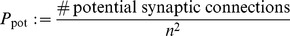
(2)


is the chance that there is a potential synapse between two neurons.

It is now tempting to apply the old memory theories for weight plasticity as well to structurally plastic networks by simply replacing 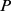 by 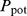. The underlying argument is that the structurally plastic network with potential connectivity 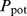 would be functionally equivalent to a structurally static network with anatomical connectivity at the same level 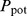 because real synapses could “migrate” to any one of the 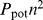 potential locations. Such an approach would be valid only if the number of required synapses does not exceed the number of actual synapses, 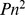. However, the question which or how many synapses are actually necessary for storing a particular memory set is usually neglected by theories for fixed networks without structural plasticity. Moreover, from such theories it is impossible to infer any temporal dynamics of structural modifications during memory formation.

**Figure 2:**
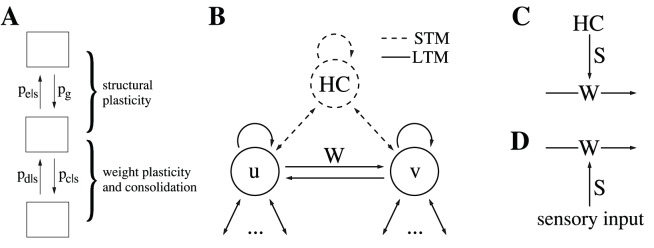
Model of structural plasticity and consolidation. **A**, State/transition model of a single potential synapse (see text for details). **B**, In the following we consider potential synapses in a network 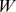, for example, connecting two cortical neuron populations 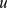 and 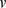. Memories correspond to associations between activity patterns 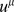 and 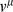. We will specifically analyze how well noisy activity patterns 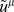 can reactivate the corresponding memories 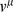 in order to estimate storage capacity. **C, D**: LTM storage (solid) by structural plasticity requires repetitive reactivation of activity patterns in cortical populations 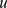 and 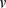 to provide an appropriate consolidation signal 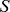 to the synapses. This may happen by repeated bottom-up stimulation (**D**) or, for episodic memories, by top-down replay (**C**) from a HC-type STM buffer (dashed). LTM = long-term memory; STM = short-term memory; HC = hippocampus.

We therefore have to introduce another type of connectivity measure that specifies how many synapses have actually been formed at time 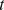 between neurons that belong to a particular memory representation. More generally, we can specify the *synapse ensemble* requested to support storage of a memory set 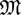 by a 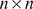 matrix 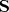. In the simplest case 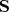 is binary where non-zero matrix entries with 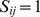 “tag” potential synapses from neuron 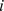 to 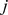 that need to be realized or consolidated for storing the memories 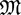 (Fig. 1B). Then with 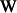 being the 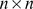 matrix of actual synaptic weights (with 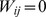 if there is no real synapse from 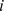 to 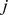), we define the *effectual connectivity of memories* 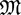 as the “overlap” of actual and requested synaptic weights, for example,
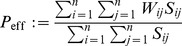
(3)


for binary synaptic weights with 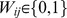 (Fig. 1B). For real-valued weights one could generalize this definition (e.g., 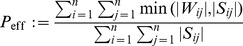 where 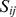 may be either binary or real-valued, specifying the “desired” synaptic weight). It is obviously 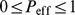 and, for eq. 3, effectual connectivity 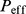 corresponds simply to the probability that a requested synapse is actually realized and potentiated (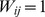). We call the matrix 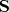 also *learning signal* or *consolidation signal* because it specifies which synapses should be potentiated or stabilized during memory consolidation. For example, simple Hebbian consolidation signals can be based on the correlations between presynaptic and postsynaptic spike activity (see next section). Such 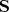 could be provided either by repeated bottom-up stimulus presentation or, in the case of episodic memory, by replay from a hippocampus-like short-term memory buffer (Fig. 2B–D). The fraction of non-zero entries in 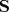 is called the *consolidation load* 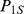. In larger networks it is typically 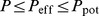 if locations of requested synapses 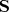 are uncorrelated to the (initial) locations of potential and actual synapses. Our main hypothesis is that the primary function of structural plasticity is to adapt network structure to the particular memories to be stored. This process corresponds to an increase in effectual connectivity 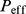 from the level of anatomical connectivity 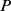 towards the level of potential connectivity 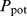 which increases storage capacity per synapse as well as space and energy efficiency of the network [47]–[49].

### 2 Model of Structural Plasticity and Consolidation

Figure 2A illustrates a minimal state model for a “potential” synapse. Here a potential synapse 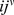 is the possible location of a real synapse connecting neuron 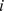 to neuron 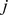, for example, a cortical location where axonal and dendritic branches of neurons 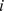 and 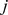 are close enough to allow the formation of a novel connection by spine growth and synaptogenesis [29]. As dendrites and axons may closely overlap at multiple locations, in general, there may be multiple potential synapses (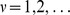) between a neuron pair 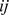. Our minimal model has only three states: A synapse can be either potential but not yet realized (state 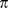), realized but silent (state and weight 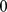), or realized and consolidated (state and weight 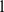). For real synapses, state transitions are modulated by the consolidation signal 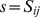.

Then *structural plasticity* means the transition processes between states 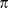 and 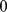 described by transition probabilities 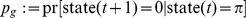 and 
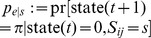
. Similarly, *weight plasticity* means the transitions between states 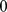 and 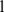 described by 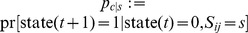 and 
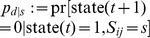
. In accordance with the diagram of Fig. 2A, the evolution of synaptic states can then be described by probabilities 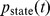 that a given potential synapse is in a certain 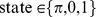 at time step 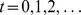,
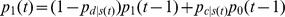


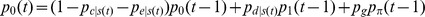


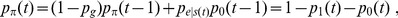
(4)where the (Hebbian) consolidation signal 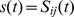 may depend on time. Note that we assume 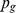 to be independent of 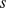 because it is unclear how to provide 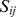 with high spatial precision 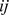 to not yet realized potential synapses. Instead, 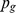 may rather be under the control of homeostatic mechanisms to keep the number of synapses or the resulting mean firing rates of a neuron at a desired level [50]. The model could easily be extended towards more biological realism by additional state transitions (e.g., from 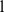 to 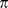 [51]), a cascade of further synaptic states [52], or graded synaptic weights [53], [54], but here the focus is on the essential properties of the interplay between structural and weight plasticity.

For the microscopic simulations of individual synapses as displayed in Figs. 4 and 6 we have used the Felix++ simulation tool [55] to implement large networks with many potential synapses and to simulate network evolution by random sampling of synaptic state variables in discrete time steps. A simple match of the simulation time scale to physiological data can be obtained from the mean lifetime of unconsolidated unrequested synapses: For 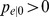 the mean lifetime is 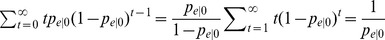 simulation steps. This may be compared, for example, to the few days lifetime reported for unstable spines in adult animals [10].

**Figure 3:**
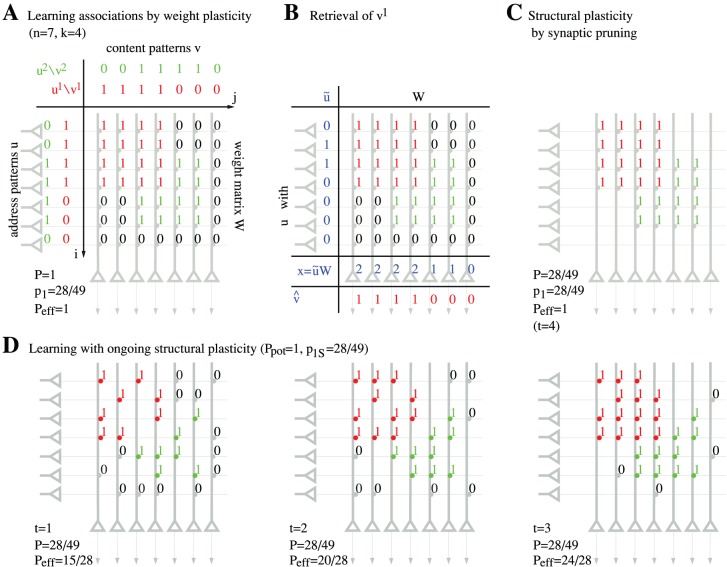
Learning in Willshaw-type associative networks. **A**, Memory storage by Hebbian weight plasticity (Eq. 5) in a fully connected network (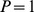). Address patterns 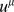 are associated to content patterns 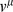 where 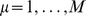 (here 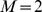). Each memory is represented by a binary activity vector of length 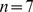 having 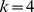 active units (which define the corresponding cell assembly). **B**, One-step retrieval of the first memory from a noisy query pattern 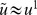 having two of the four active units in 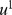 (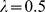). Here 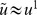 can perfectly reactivate the corresponding memory pattern in population 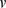 (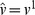) applying a firing threshold 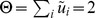 on dendritic potentials 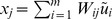. **C**, As a simple form of structural plasticity, silent synapses can be pruned *after* learning. The resulting network has only 28 (instead of 49) synapses corresponding to a lower anatomical connectivity 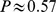, whereas the effectual connectivity is still 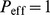. Thus, pruning does not change network function, but increases stored information per synapse. **D**, Ongoing structural plasticity can similarly increase storage capacity during more realistic learning in networks with low anatomical connectivity (here 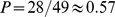). During each time step 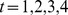, Hebbian weight plasticity potentiates and consolidates synapses 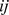 with non-zero consolidation signal 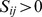 (which equals 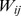 of panel A), whereas the remaining silent synapses are eliminated and replaced by new synapses at random locations. Note that the resulting network at 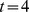 is the same as in panel C.

**Figure 4:**
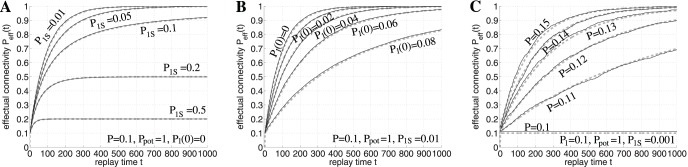
Increase of effectual connectivity during memory consolidation with ongoing structural plasticity. Each curve shows the evolution of effectual connectivity 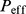 as a function of time 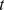 for different parameters 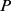 (anatomical connectivity), 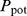 (potential connectivity), 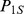 (consolidation load), and 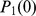 (fraction of initially consolidated synapses). Data are from single microscopic network simulations (solid black; cf. Eq. 4; network size 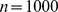) and macroscopic theory (dashed gray; Eq. 11). See Table 1 for further simulation parameters. **A**: 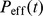 for different consolidation loads 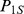 and constant 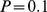, 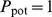, 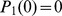. **B**: 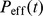 for different fractions of initially consolidated synapses 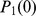 and constant 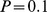, 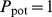, 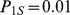. **C**: 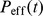 for different anatomical connectivities 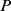 and constant 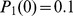, 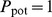, 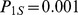.

On the network level we use corresponding *macroscopic* variables 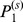, 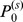, and 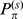 defined as the fraction of neuron pairs that have a potential synapse in a certain state and receive a certain consolidation signal 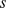. From this we can derive the connectivity variables defined in the previous section, in particular, 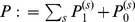 and 
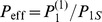
 for binary 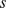 (see Sect. Mathematical Analysis I for details). In most simulations of (adult) memory processes (Figs. 4,3D,6), we have assumed that the rates of synapse generation and elimination are in homeostatic balance to maintain either a constant anatomical network connectivity 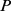 or a constant number 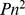 of actual synapses.

**Figure 5:**
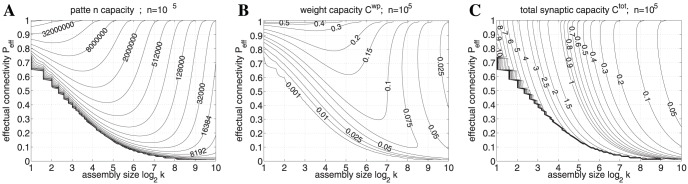
Storage capacities for a finite Willshaw network having the size of a cortical macrocolumn (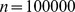). **A,** Contour plot of pattern capacity 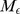 (number of stored memories) as a function of assembly size 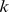 (number of active units in a memory vector) and effectual network connectivity 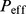 assuming output noise level 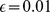 and noise-free input patterns (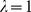, 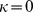). **B,** Weight capacity 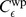 for the same setting as in panel **A**. **C,** Total storage capacity 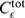 including structural plasticity for the same setting as in **A**. Note that even modest increases of 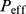 can strongly increase storage capacity, in particular for sparse neural activity (small 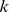) [82]. All data computed from Gaussian approximation of dendritic potential distributions (see appendix II. 2).

**Figure 6:**
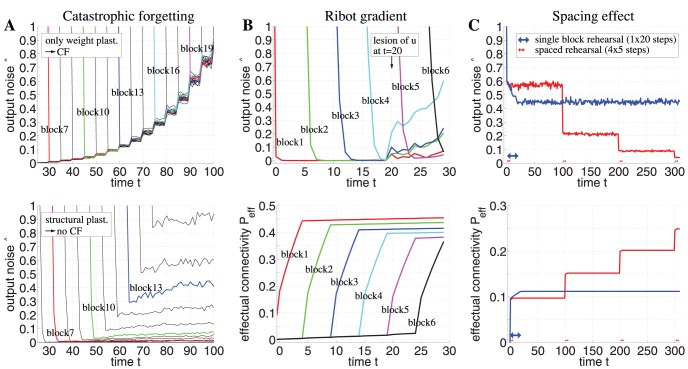
Simulation of catastrophic forgetting, Ribot gradients, and the spacing effect. **A**, Networks without structural plasticity suffer from catastrophic forgetting (top), but networks with structural plasticity do not (bottom). Plots show output noise 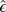 over time 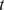 simulating networks of size 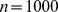 and activity 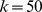 storing 25 memory blocks one after the other (only the interesting part between storage of blocks 6 and 21 are visible). Each curve (with a distinct color) corresponds to 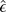 for noisy test patterns of a particular memory block with 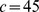 correct and 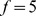 false active units. The steep descent of each curve corresponds to the time when the Hippocampus started to replay the corresponding memory block for 5 time steps. **B**, Networks employing structural plasticity show Ribot gradients after a cortical lesion (top) due to gradients in effectual connectivity (bottom). The lesion was simulated by deactivating half of the neurons in population 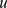 at time 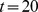. **C**, Networks employing structural plasticity reproduce the spacing effect of learning. In the first simulation (blue) novel memories were rehearsed once for 20 time steps (blue arrow at 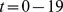). In a second simulation (red) the same total rehearsal time was “spaced” or distributed to four brief intervals of five steps each (red arrows at 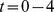, 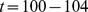, 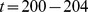, and 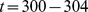). Here the network achieves a higher effectual connectivity 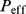 (bottom) and less retrieval noise 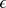 (top). See Sections 2, 3 and Table 1 for further details and simulation parameters.

The relation between synapse and network variables is non-trivial in general because there may be multiple potential synapses 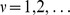 per neuron pair 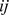 (see Sect. Mathematical Analysis I.1), for example around 5–10 between two connected neighboring cortical neurons [56]–[60]. Nevertheless, we argue that even our simple binary model with only a *single synapse* per connected neuron pair bears significant biological relevance because it has been reported that the number of actual synapses per connected neuron pair and also the total synaptic weight is surprisingly similar across neurons (see discussion section; cf. [59], [61]). Therefore, we have analyzed this simple model to obtain the results presented below and in Section 6 (see Figs. 4–5). To improve biological realism of our simulation experiments in Section 7 (Fig. 6), we have tested our ideas also with a second model variant that allows *multiple synapses* per neuron pair, where each of the 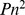 actual synapses of the network can be allocated to one of the 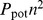 potential locations independently of other synapses. Additional simulations (not shown) have indicated that both model variants yield qualitatively very similar results unless the replay time for a given consolidation signal was very long. Then the second model variant tended to accumulate all available synapses at the locations specified by the consolidation signal such that neuron pairs were connected by a large number of synapses.

### 3 Models for Memory Storage and Retrieval

The model presented so far is of general relevance for any neural theory of memory, because it is independent of any specific mechanisms for memory storage and retrieval: Any learning and storing mechanisms are only implicitly conveyed by the learning signal 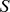 that “tags” potential synapses for later consolidation. Similarly, memory recall is not directly described in the model so far. Rather, our theory describes effectual connectivity 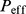 which is closely linked to retrieval performance for a given memory set. To explain this link and to allow a more quantitative performance evaluation, the next section instantiates and analyzes our model within a common neural network framework of memory storage and recall.

A particularly simple memory model based on Hebbian learning of binary synapses is the *Steinbuch* or *Willshaw model* [26], [44], [62]. In the general *hetero-associative* setup (Fig. 3A), memories correspond to binary spike activity vectors 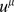 and 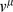 stored in a synaptic connection 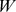 linking two neuron populations 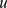 and 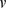. By choosing the *auto-associative* setup with identical 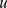 and 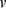, the Willshaw model can be applied as well to model memory processes in local recurrent connections (cf. Fig. 2B). The average number 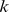 of one-entries in an activity vector is called *pattern activity* and corresponds to the mean size of local Hebbian cell assemblies in populations 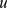 and 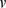. After *storing* a set of 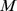 memory associations in a network without structural plasticity, the weight of an actual synapse connecting neuron 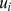 to neuron 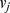 is
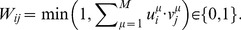
(5)


Note that a synapse in the Willshaw model is actually a special case of our model of a potential synapse because Eq. 5 instantiates Eq. 4 for 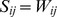, 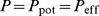, 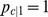, and 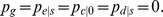


*Memory retrieval* means the re-activation of a previously stored content pattern 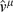 in neuron population 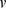 following the activation of a (noisy) address pattern 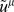 in population 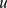. The simplest retrieval procedure is “one-step retrieval” with adaptive threshold control [63]. Specifically, an input pattern 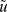 is propagated synchronously from population 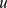 to population 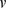 as illustrated in Fig. 3B. Then dendritic potentials of the neurons in population 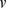 are given by simple vector-matrix-multiplication, 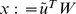, and the retrieval output 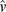 is obtained from 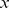 by applying a vector of spike thresholds 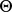,
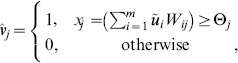
(6)where 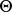 is chosen to obtain close to 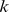 active units in 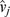. We can then evaluate retrieval quality by estimating the *output noise* level



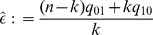
(7)defined as the mean Hamming distance 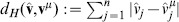 between retrieval output 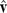 and the original memory 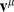 normalized to the cell assembly size 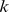. Here 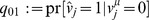 and 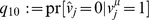 are component error probabilities. Similarly, we can define input noise 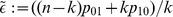 as the normalized Hamming distance between input pattern 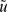 and the original address memory 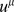. We will also express input noise in terms of parameters 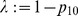 (completeness) and 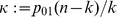 (add noise).

We have used one-step retrieval for some of our experiments (Fig. 5) because it is most easy to analyze, for example, for estimating the memory capacity of a single network (see below). However, for the investigation of memory phenomena, there exist more realistic retrieval methods that are based on spiking neurons and iterative (gamma range) oscillatory activity propagation [64], [65]. As such models are computationally very demanding, in particular, when simulating longer time intervals in the range of months to years, it is more favorable to use simple iterative extensions of one-step retrieval [27], [63], [66], [67]) that can still mimic many relevant properties of the realistic models.

In particular, iterative retrieval avoids the most serious limitation of one-step retrieval, that is, the lack of a sufficient attractor behavior: High output noise after one-step retrieval does not exclude perfect retrieval after iterated retrieval steps. In fact, as long as the output noise level after the first step is smaller than the input noise level, the iterative retrieval procedure is likely to reduce output noise to zero in subsequent retrieval steps. As a consequence, for *individual* memories, the relation between input and output noise will be much steeper if using the iterative models: A memory pattern can be retrieved either perfectly or the number of component errors is very high. Still, one-step retrieval is useful by providing lower bounds (because of its suboptimality) and upper bounds (assuming zero input noise) of the true storage capacity.

For our long-term simulations of memory phenomena (Fig. 6) we have therefore extended the Willshaw model in two ways: First, similar as illustrated by Fig. 2B, we have included also Willshaw-type auto-associative connections in addition to the hetero-associative link from 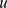 to 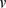 in order to account for the rich recurrent connectivity of cortex and to enable iterative refinement of retrieval outputs. Second, we have implemented an *iterative retrieval* procedure as follows (cf. [63]): In an initial step, the input pattern 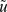 is propagated through the hetero-associative connections from 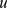 to population 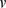, in which the 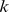 neurons with the largest dendritic potentials become active, resulting in a preliminary retrieval result 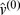. In similar further steps, this preliminary result was then iteratively propagated through the auto-associative network of population 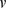 yielding refined retrieval outputs 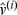 for 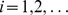 (where all recurrent connections to 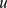 were inactivated). Typically, a small number of iterations was sufficient to obtain stable outputs. For evaluation of output noise 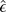 we used the activity pattern 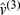 after 3 iterations and compared it to the original memory pattern 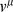 to estimate component error probabilities 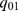 and 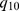 (see Eq. 7).

For the simulations involving structurally plastic networks and long-term consolidation (Fig. 6) we have divided the overall memory set into multiple blocks 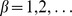 each containing several individual memory patterns. Each memory block defines a consolidation signal 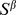 that is identical to the Willshaw matrix (Eq.5) obtained from the corresponding subset of memories. Thus, memory blocks are consolidated one after the other, each for a certain number of simulation steps, by reactivating the corresponding activity patterns in populations 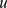 and 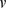 to mimic either hippocampal short-term storage and top-down replay (Fig. 2B,C) or repeated bottom-up rehearsal of the corresponding memories (Fig. 2B,D). Fig. 6 shows simulations with structural plasticity in the connection 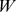 linking 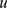 to 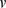. By contrast, the recurrent connections within 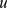 and 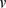 were prewired without any structural plasticity and auto-associatively stored the individual patterns 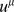 and 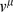 with a fixed connectivity (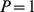 for Fig. 6A, upper panel; 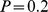 for Fig. 6A, lower panel; 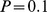 for Fig. 6B,C). Table 1 summarizes the remaining simulation parameters.

**Table 1:** Simulation parameters.

Figure No.	synapse model	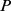	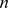	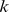	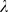	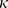	#blocks	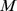 /block	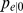
4A	single	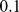	1000	10	-	-	1	100–6931	0.1
4B	single	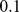	1000	10	-	-	1	100	0.1
4C	single	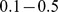	1000	10	-	-	1	100	0.1
6A, upper	multi	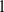	1000	50	0.9	0.1	25	12	0
6A, lower	multi	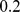	1000	50	0.9	0.1	25	4	1
6B	multi	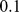	1000	50	0.9	0.1	6	4	1
6C	multi	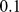	1000	50	0.9	0.1	1	20	0.01

Model parameters for the simulations shown in Figs. 4 and 6. All simulations used 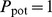, 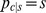 and 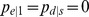 for 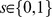 and a homeostatic setting maintaining a constant anatomical connectivity 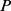 where the number of eliminated synapses per time step was equal to the number of generated synapses, i.e., 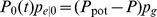, where 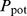 is the potential connectivity and 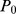 is the fraction of unconsolidated silent synapses at time 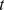. Synapse model: “single”  =  at most a single synapse per neuron pair; “multi”  =  multiple synapses per neuron pair are possible. 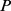 is anatomical connectivity of the synaptic projection from population 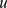 to 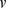. 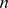 is population size of 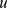 and 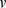. 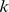 is cell assembly size (i.e., number of one-entries in memory patterns). 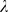 and 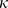 denote completeness and add noise in input patterns 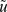 (i.e., 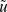 has 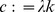 correct and 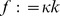 false one-entries). # blocks is number of memory blocks that are subsequentially stored. 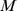/block is the number of memories per memory block. 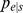, 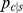, 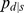 are the state transition probabilities of the (potential) synapses.

### 4 Definitions of Storage Capacity

The *storage capacity* is the amount of information (in bits) that a neural network can store (and retrieve) per synapse. There are two contributions to the total capacity 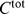 of a synapse,
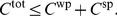
(8)


First, the *weight capacity* 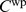 is the information stored by modification of the synaptic weight for a fixed network structure. (a more general definition could as well include any other modifications of synaptic *state* variables such as synaptic transmission delay). Second, the *structural capacity* 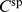 is the information stored by selecting an appropriate target location for a synapse with fixed weight. We would like to evaluate storage capacity at a limited small output noise level 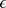 (see Eq. 7): The “stored information” can then be computed from the *pattern capacity* 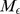 defined as the maximum number of memories that can be stored at noise level 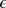, whereas the *weight capacity* 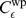 is the stored information normalized to the number of synapses in a static network (no structural plasticity) with connectivity 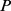,
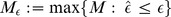
(9)

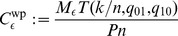
(10)


where 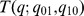 is the transinformation (or mutual information) when transmitting independent memory components 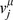 (with 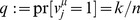) over a binary channel (with transition probabilities 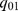 and 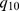 as in Eq. 7) and receiving 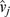 (for details see appendix A in [16]). In general, it is difficult to disentangle the two contributions 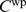 and 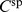. Thus, in the results section we will compute the total capacity 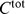 for some special cases.

## Results

### 5 Structural Plasticity Increases Effectual Connectivity

In the previous section we have introduced effectual connectivity 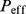 as a measure of how well a given set of memories is stored in a synaptic network. Without any structural changes of the network, 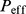 will obviously remain constant, for example, at the level of anatomical connectivity 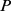 for novel memories that do not correlate with the current network structure. It is therefore more interesting to investigate the dynamics of 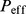 during phases of ongoing structural plasticity. For consistency with experimental observations it seems most reasonable to focus on a parameter range where structural plasticity operates on a slower time scale than Hebbian-type weight plasticity (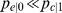), but on a faster time scale than the lifetime of stable consolidated synapses (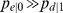).

It is indeed possible to analyze our model in such a parameter regime: In Sect. Mathematical Analysis I.2 we compute the temporal evolution of effectual connectivity during consolidation of a novel memory set under the following simplifying assumptions: 1) Large networks with 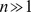 such that all macroscopic variables 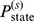 are close to their means; 2) at most a single synapse per neuron pair; 3) binary consolidation signal 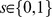; 4) new memories specified by 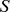 are independent of initial network structure and any old memories; 5) immediate consolidation with 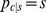; 6) 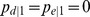; 7) 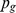 and 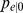 in homeostatic balance such that 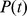 is constant. Then effectual connectivity for a new set of memories increases from 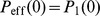 before any learning starts to
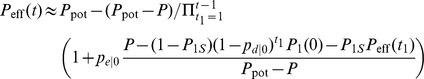


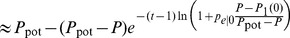
(11)


assuming that 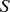 is provided at each time step 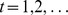 (e.g., by memory replay) and 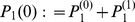 is the fraction of initially consolidated synapses (corresponding to old memories). The second approximation additionally presumes 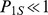 and 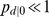. Thus, convergence of 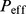 towards 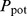 requires 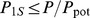 (for 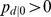) or 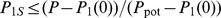 (for 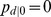). Also note that during the first consolidation step there is a quick increase from 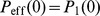 to 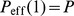 followed by a much slower increase towards 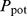 in the subsequent steps. Section 7.1 relates this behavior to the spacing effect as a possible explanation why several brief learning sessions are generally more effective than a single long session.

**Figure 7:**
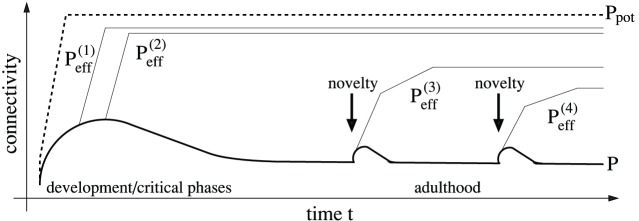
Sketch of network connectivity reflecting lifelong structural plasticity. During development anatomical connectivity 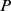 (thick solid) quickly increases reaching a peak level (around 2–3y in humans), where the initial increase is followed by a short period of stable connectivity (until age 5y in humans), a phase of significant decrease of connectivity until puberty, and finally a phase of stable connectivity during adulthood [14], [51], [77]. Recent experiments suggest a temporary novelty-driven (thick arrows) increase of connectivity during adulthood [23], [68], [116]. Our model of structural plasticity predicts that learning is fastest for high levels of anatomical connectivity and structural plasticity. Thus, memories acquired during early phases can reach higher levels of effectual connectivity (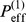,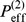; thin solid lines) compared to memories acquired during later phases (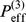,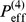). The resulting gradients in effectual connectivity can explain various memory phenomena (see Section 7 for details).

Figure 4 shows that the approximations accurately predict microscopic model simulations. Consolidation becomes slower for larger consolidation loads 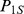 which limits maximal storage capacity (panel A; see Section 6). Similarly, consolidation becomes slower for increasing fractions 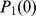 of initially consolidated synapses (panel B). As 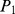 will correlate with the number of previously consolidated memories and, thus, with age, this implies that memory consolidation should be faster in young compared to old subjects, even if the anatomical connectivity 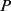 would be constant over lifetime. Moreover, the corresponding gradients in 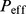 resulting after a fixed number of consolidation steps can be related to gradients in memory performance in graded retrograde amnesia (Section 7.2) and the absence of catastrophic forgetting (Section 7.1). Finally, panel C shows that even slight increases in anatomical connectivity (as reported after learning new concepts or tasks [68]; cf. Fig. 7) can strongly speed-up memory consolidation if a large proportion of synapses are in the consolidated state (as expected for adult networks after synaptic pruning [14], [15]).

Our analysis and further simulations (data not shown) reveal that the described increase of 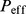 is very stable and occurs for virtual any plausible configuration of model parameters. Before we discuss the mentioned memory phenomena in more detail, the following shows that, by increasing 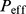, structural plasticity can store much more information per synapse than Hebbian-type weight plasticity.

### 6 How Much Information can a Synapse Store?

It is a well-known result of information theory [69] that optimally coding an entity taken at random from a set of 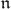 different entities takes 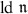 bits of information [69] (where 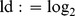). From this we can derive simple upper bounds for the maximal information that a synapse can store by counting the number of possible synaptic states, i.e. the number of possible weights and locations, that can be realized by weight plasticity and structural plasticity, respectively. The resulting upper bounds for weight capacity 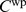 and structural capacity 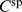 are
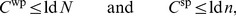
(12)


assuming that weight plasticity can choose one out of 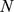 possible discrete weights for an individual synapse, and structural plasticity can choose between 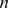 targets where to grow a novel synapse. These bounds could trivially be reached by an ideal observer that has direct access to synaptic attributes (i.e., weights and locations). However, here we are rather interested in how much information a synaptic network can store *and* safely retrieve employing biologically plausible mechanisms. In particular, we have to measure the amount of retrieved information from plausible neural output variables such as spikes or mean firing rates. For this it is necessary to link our theory to concrete neural network models of memory storage and retrieval, such as Willshaw and Hopfield-type models ([26], [27], [45], [70], [71]; see section 3).

Our theory yields the surprising result that the weight capacity 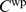 in the brain might actually be negligible compared to structural capacity 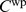. First, it is well understood that weight capacity of biologically plausible memory models is limited by hard theoretical bounds suggesting 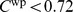 bit per synapse even for an infinite computing precision with 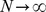 [27], [45], [46], [72], [73]. Second, due to noisy transmission characteristics and various adaptation mechanisms, real synapses are likely to have a rather small number of functionally distinctive states, perhaps 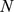 being on the order of ten or even binary [74]–[76]. Third, unlike 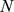, the number of potential targets 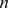 may actually be very large in the brain: For example, for a cortical neuron 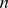 is on the order of 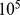 corresponding to the number of neighboring cells within the same macrocolumn [24], and the number of targets 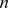 may be even much larger because each neuron may have a large number of functionally distinct dendritic compartments [28]. Fourth, it has been recently shown that the upper bound of structural capacity can be tightly reached for synaptic pruning following learning in completely connected networks [16], [53].

Before generalizing these results to ongoing structural plasticity in sparsely connected networks, let us first re-analyze the classical Willshaw model (without structural plasticity) as illustrated in Fig. 3A,B. There, synaptic weight plasticity follows a simple binary Hebbian rule (Eq. 5). Due to 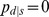 (cf. Eq. 4) the fraction of consolidated synapses 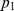 increases monotonically with 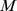 until it reaches a maximal value 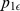 beyond which the output noise 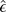 exceeds the tolerable level 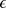. Some theory presented in Sect. Mathematical Analysis II.1 shows that the corresponding pattern capacity 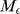 crucially depends on 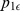: For networks of size 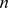, randomly generated cell assemblies of size 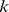, and input noise with 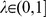 and 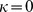, it is (see text below Eq. 28 in Sect. Mathematical Analysis II.1)
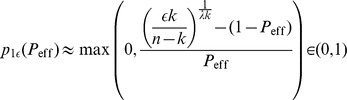


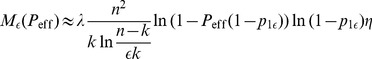
(13)where factor 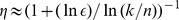 comes close to one for large networks. Multiplication by the stored information per memory and dividing by the number of synapses gives the well known weight capacity of the Willshaw model (see Sect. Mathematical Analysis II. 1),



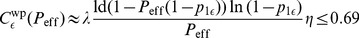
(14)where the upper bound 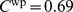 bps can be reached for large networks, 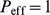, 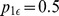, sparse activity 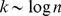, and zero input noise with 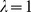.

In previous works on structural plasticity we have focused on *synaptic pruning* of silent synapses after learning all memories in a *fully connected* network (Fig. 3C). Here we extend these results to networks with incomplete (“diluted”) connectivity and ongoing structural plasticity. Let us first consider synaptic pruning which has been described as one of three phases during brain development (e.g., in humans, synaptic density increases until age of 2–3 years, then remains stable until 5 y, then decreases until puberty and remains relatively stable during adulthood; cf. [14], [51], [77]; see also Fig. 7):

1. Synaptic overgrowth: The synaptic generation rate is much larger than the elimination rate, 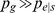, such that anatomical connectivity 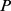 can come close to potential connectivity 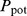.
2. Critical consolidation phase: Weight plasticity potentiates and consolidates useful synapses that support memory contents specified by the consolidation signal 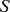, e.g., 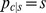, 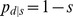.
3. Synaptic pruning: Useless synapses are eliminated, e.g., 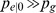 (cf. Fig. 3C).

Because only a fraction 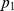 of the synapses survives phase three, the total storage capacity at maximal 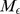 (where 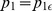) is obtained from renormalizing Eq. 14,
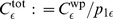
(15)


Using 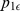 from Eq. 13 reveals that 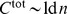 for sufficiently small cell assembly sizes 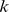 (see Sect. Mathematical Analysis II. 1). Thus, the Willshaw model with structural plasticity comes close to the information-theoretic capacity bound (Eq. 12). We have shown elsewhere that 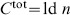 can be reached tightly with much weaker assumptions on cell assembly sizes and effectual connectivity by inhibitory implementations of the Willshaw model [46], [78] and both excitatory and inhibitory implementations of Bayesian networks with discrete synaptic weights [53], [54], [79].

Unlike in development, during adulthood anatomical connectivity is stable. This means that ongoing generation and elimination of synapses must be in homeostatic balance such that the total number of synaptic connections remains approximately constant over time [14], [80], [81]. In the following we show that ongoing structural plasticity during adulthood can reach the same high storage capacity as during development, although this process may require significantly more time. The basic idea is that the three developmental processing phases (synaptic generation, consolidation, and elimination) run in parallel during each time step 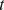. For example, by choosing the synapse parameters.
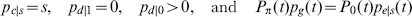
(16)the anatomical connectivity 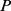 remains constant and, in essence, all actual synapses “migrate” to the locations 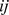 specified by the consolidation signal 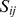 (cf. Fig. 3D). *IF* 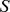 specifies *all* memories to be stored, 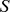 is applied during each time step, and the consolidation load 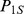 is sufficiently large such that 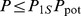, *THEN* memories will be stored at effectual connectivity 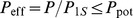, there will be no silent synapses left, and the resulting total capacity 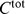 is given by Eq. 15. In particular, for 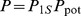 the resulting network will be identical as for developmental learning described before (see Fig. 3D and compare to Fig. 3C). This shows that also adult learning in structurally plastic networks with constant low anatomical connectivity can reach the information theoretic bound 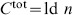 (see Eq. 12).

In the following we apply our theory to networks with biologically relevant parameters. For example, a typical network size may correspond to a cortical macrocolumn of size 1 mm^3^ containing about 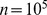 neurons and relatively dense recurrent connections with an anatomical connectivity of about 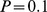 [24], [25]. Then we can estimate potential connectivity 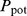 from experimental measurements of the *filling fraction* 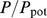 defined as the fraction of potential synapses that is actually realized (i.e., in state 0 or state 1). For typical 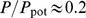 [29], structural plasticity of dendritic spines alone may account already for 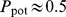 within a neocortical macrocolumn. The corresponding storage capacities are depicted in Figure 5. Note that without structural plasticity (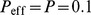) the storage capacity remains tiny, e.g., 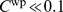 for 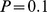. In particular, sparse activity patterns [82] cannot be stored at a low connectivity, e.g., 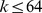 requires 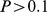 to stabilize even a single memory pattern.

By contrast, networks employing structural plasticity with potential connectivity 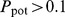 can have a large total capacity 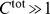. Interestingly, 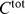 increases with decreasing connectivity. Thus, even slight increases of effectual connectivity towards 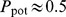 can strongly increase number of stored memories (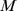) and even maximize stored information per synapse (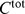). Note that an increase in 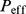 during consolidation would also allow a simultaneous decrease of activity 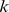 to maximize capacity. This means that consolidation involving structural plasticity and sparsification will move the “working point” from the lower right towards the upper left in the contour plots of Fig. 5. Thus, by emulating high effectual connectivity, structural plasticity may also support the sparsification of memory representations [82]–[85] and stabilize small cell assemblies that would appear unstable for a fixed low connectivity [86], [87].

The following sections show that structural plasticity, in addition to increasing storage capacity, can explain several well known memory phenomena in the brain much better than previous theories.

### 7 Relevance of Structural Plasticity for Memory Phenomena

#### 7.1 Absence of Catastrophic Forgetting

Artificial neural networks such as multi-layer-perceptrons are well known to suffer from what was called catastrophic forgetting (CF) or the stability-plasticity dilemma [36], [88]–[91]. It is the problem that optimizing synaptic weights to store a set of new memories will deteriorate or even destroy previous memories. Freezing synaptic weights can avoid CF, but it also hampers the ability to learn new memories.

Another form of CF has been described for Hopfield-type network models of associative memory [92]. Here CF means that a neural network with fixed structure can almost perfectly store and retrieve memories until the maximal pattern capacity 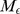 is reached. However, exceeding 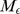 even by a few additional patterns can destroy the ability to retrieve any of the memories. The same problem occurs when increasing the number of stored memory patterns in the Willshaw-type binary learning models (Fig. 3A, B), even before the point where *all* synapses are uniformly potentiated and therefore have lost specific information about the memory patterns.

CF poses problems for technical applications, but also for modeling memory processes because it does not normally occur in our brains. It has been argued that the capacity of the brain might just be too large for running into CF during a normal lifetime. In addition, several alternative solutions have been suggested. For example, many previous approaches suggested to have an additional hidden neural layer (e.g., between populations 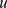 and 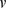) in which a new node is allocated for each new input that deviates significantly from previously stored items. The underlying idea is that in a modular organization, separate subnetworks (comprising different subsets of neuron in the intermediate layer) could be trained independently to represent different memories or categories. Such approaches include ART-type architectures [90], emergent category-specific modularity [93], hard-wired modularity [94], and also ideas involving grandmother cells [95] or, in technical terms, look-up-tables [16]. One problem with these approaches is that some high-level mechanism is required for allocating or even generating new neurons in the intermediate layer. However, in most parts of the adult brain, there is little evidence for structural plasticity involving neuron genesis. But without neurogenesis such models also predict catastrophic forgetting at a later time unless plasticity is explicitly switched off after all neurons in the intermediate reservoir have been allocated. Alternative high level mechanisms for preventing CF involve pseudo-rehearsal using self-generated training stimuli from previously learned memories [92]. In the following we are focusing on solutions to CF that can be built at the level of synapses. For example, palimpsests network models [96]–[98] assume a slow decay of synaptic weights (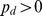) to prevent approaching the network's capacity limit, however, are not plausible for long-term storage in neocortex. Similarly, synaptic cascade models [52] introduce several consolidated states 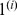 with decreasing decay rates 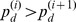. However, this cannot prevent exponential decay of memories unless the lowest decay rate is zero causing again CF.

A novel role in preventing CF can be attributed to structural synaptic plasticity: Fig. 6A illustrates simulation experiments investigating consolidation of multiple memory blocks each consisting of several novel memories. Each memory block is stored in the hippocampus and replayed to neocortical cell populations 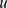 and 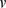 for a certain time as described before (Fig. 2B, C). As expected, without any structural plasticity (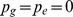) the network exhibits CF when approaching the capacity limit (upper panel). In contrast, CF is absent in networks with structural plasticity (lower panel). In this case, early stored memories remain stable all the time whereas the ability to store novel memories fades gradually when approaching the capacity limit. This behavior is more consistent with aging effects of human memory [99] and results from the fraction of consolidated synapses steadily increasing with age and the number of stored memories. Correspondingly, the fraction of unconsolidated synapses participating in structural plasticity gradually decreases with age as observed in neurophysiological experiments [21].

More precisely, for memories stored with a certain effectual connectivity 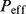, structural plasticity can prevent CF only if the filling fraction is below the maximal fraction of consolidated synapses at the capacity limit, 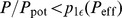 (see Eq. 13). This condition ensures that the total number of synapses, 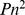, is smaller than the maximally allowed number of consolidated synapses, 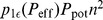, at the network's capacity limit. If fulfilled, the network can never exceed its capacity limit which effectively prevents catastrophic forgetting. Brain networks could satisfy this condition by maintaining a constant (or slowly decreasing; cf, Fig. 7) anatomical connectivity 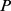 and by adapting cell assembly size 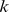 appropriately in relation to network size 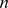 and some target effectual connectivity 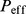. Thus, early memories can be consolidated up to some target connectivity 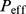 which depends on the replay time per memory block. However, at least if replay time per memory remains constant over lifetime, then for later memories 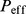 and 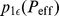 will decrease gradually with the decreasing fraction of available structurally plastic synapses, 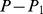 (see Fig. 4B). Therefore, the ability to learn new memories will begin to fade when 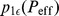 approaches 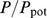.

#### 7.2 Ribot gradients in retrograde amnesia

Patients with lesions of the hippocampus or neighboring neocortex in the medial temporal lobe often suffer from graded retrograde amnesia [38], [40], [100], [101]. This form of memory loss shows characteristic “Ribot gradients” describing the tendency that recently stored memories are more likely to be lost than remote memories acquired at an earlier time. Simple palimpsests-type memory models (with 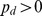) cannot account for these findings, in fact they predict the reverse effect [96]–[98].

A body of previous work has proposed that the lesions may disrupt cortico-hippocampal memory replay and, as a result, recent memories disappear because they are not sufficiently consolidated in intact neocortex [34], [35], [38], [39], [102]–[104]. According to such models, the cause of Ribot gradients is a gradient in accumulated replay and consolidation time [102], [104].

In one of the models [102], for example, replay is controlled by a random walk over the attractor-landscape in Hopfield-type networks where each stored memory 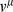 corresponds to one of the attractors. After acquiring the 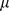
th memory, each memory obtains an 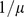 share of replay time. It is concluded that Ribot gradients occur because early memories (smaller 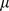) can accumulate a larger total consolidation time of about 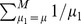 than recent memories, resulting in a larger strength of the memory trace.

Such models predict either that memories would be replayed and consolidated for an unlimited time [102] or that Ribot gradients would occur only for memories acquired during a limited time interval before the lesion occurred [104]. Although there are not yet final experimental answers [34], [105], both predictions may be in conflict with evidence that novel memories are buffered and replayed by the hippocampus for a limited time only [34], [38], [39] and that, depending on the lesion size, graded amnesia can reach back to early childhood [38].

Synaptic learning based on structural plasticity offers an alternative explanation for Ribot gradients without relying on unlimited memory replay (Fig. 6B). According to our model, the substrate of Ribot gradients are gradients in effectual connectivity 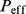 instead of (or in addition to) gradients in accumulated consolidation time. Even with constant replay time per memory, remote memories are stored with a larger 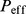 than recent memories, for the very same reasons that explained the absence of catastrophic forgetting. Correspondingly, output noise 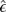 will be largest for most recent memories. During normal operation 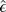 is sufficiently low to accurately retrieve both remote and recent memories. However, cortical or hippocampal lesions will increase noise-levels such that memories get lost for which 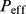 is below some critical value, or equivalently, that have been stored after some critical time point.

#### 7.3 Spacing effect

Another interesting feature of memory is that learning new items is more effective if rehearsal is spaced over time compared to single block rehearsal [41]–[43], [106]. For example, learning a list of vocabularies in two sessions each lasting 10 minutes turns out to be more effective than learning in a single session lasting 20 minutes. This so-called spacing effect is remarkably robust and occurs in many explicit and implicit memory tasks in humans and many animals being effective over many time scales from single days to months.

Previous cognitive models attributed the spacing effect either to deficient processing of repeated items during single block rehearsal [107] or to improved storage by exploiting context variability between spaced rehearsal sessions [108]. Typically, these explanations presumed specific high-level structures and mechanisms of memory systems including attention, novelty, and context processing. Although detailed modeling of memory systems may be required to explain specific properties in particular memory tasks, the ubiquity of the spacing effect suggests a common underlying mechanism at the cellular level. We propose that structural plasticity in sparsely connected neural networks is such a mechanism.

Figure 6C shows that structurally plastic networks reproduce the spacing effect naturally when learning a new set of memories in a similar protocol as described for the previous simulations (only here the memory replay should be interpreted more generally as rehearsal, not necessarily generated by the hippocampus). In the first simulation (blue) the memories are rehearsed in a single long time block, while in the second simulation (red) rehearsal is spaced over several shorter blocks such that total rehearsal time is equal for both simulations. For spaced rehearsal the resulting effectual connectivity 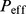 of the memories turns out to be much higher and, correspondingly, the output noise 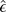 much lower than for single block rehearsal.

Further simulation experiments (not shown) have indicated that the spacing effect induced by structural plasticity is very stable. Similar to the psychological experiments, it is remarkably difficult to find conditions without spacing effect. In essence, the spacing effect occurs if weight plasticity is faster than structural plasticity and if consolidated synapses are more stable than silent synapses (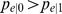). Both properties are strongly supported by experiments [4], [10], [21], [109]. In this case, our theory predicts that even in brief rehearsal sessions Hebbian plasticity can quickly consolidate all available synapses useful to store a set of memories. Thus, instead of continuing a rehearsal session, it is better to wait until structural plasticity has grown additional useful synapses that can then be consolidated in a brief second rehearsal session. As a consequence, spacing effects will necessarily occur whenever learning in the brain depends on structural plasticity. Interestingly, our model with structural plasticity can also quantitatively reproduce long-term spacing effects as recently observed in psychological experiments that investigated optimal spacing intervals to maximize memory retention [110], [111].

## Discussion

One important limitation in the brain seems to be the number or density of functional (non-silent) synapses, both for anatomical and metabolic reasons. For example, the number of synapses per cortical volume is remarkably similar across different species [112], and theoretical considerations suggest that the energy consumption of the brain is dominated by the number of postsynaptic potentials or, equivalently, the number of functional non-silent synapses [47]–[49]. In face of these limitation, it might be beneficial that learning in brain circuits “moves” synapses to computationally useful locations [16], [31], [53], [113].

To get a quantitative grip of these ideas we have introduced the concept of effectual connectivity, a macroscopic measure for how useful network structure is for memory storage. Structural plasticity can increase effectual connectivity while keeping the anatomical connectivity (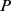) at a low constant level. This has been analyzed for a simple model of structural plasticity assuming the following three basic mechanisms: (1) blind synaptogenesis, (2) consolidation of useful synapses, and (3) elimination of irrelevant synapses. Further, we have focused on the most plausible parameter range where structural plasticity (1,3) operates on a slower time scale than weight plasticity and consolidation (2), but the lifetime of consolidated synapses is long compared to the turnover of unstable synapses (see Section 2 and Section 5 for details; cf. [4], [10], [21]). In our current model implementation we identify strong synapses with stable synapses (weight and state 1) as well as weak synapses with unstable synapses (weight and state 0). This contrasts with some experimental results suggesting that silent synapses could be quite stable [114] whereas even strong synapses could be eliminated, for example, during development [51]. Such findings may be explained by the probabilistic nature of state transitions in our synapse model or a dissociation between synaptic strength and stability, perhaps including a cascade of several different stable and unstable states [52].

Our model is applicable to learning during development, as well as during adulthood (Fig. 7). During development the three mechanisms appear to dominate different phases separated on a large time scale of years [14]–[16], [51], [77], [115]. Still, on a smaller time scale of days or months [20], [21], [23], ongoing structural plasticity, involving the three mechanisms simultaneously, could control the anatomical connectivity to be approximately constant (see Eq. 16). Such homeostatic regulation of generation and elimination of synapses is even more evident during adulthood where the anatomical connectivity appears almost stable over several decades [14], [51], [77]. However, recent experiments demonstrate that there can be novelty-driven excursions from homeostatic balance on the time scale of several days in specific cortical areas of the adult brain, for example, during learning of motor memories [23], [68], [116]. This phenomenon can be understood within our modeling framework as a different control strategy of the anatomical connectivity, one which is driven by learning load. Specifically, in instances of high learning load, up-regulating the anatomical network connectivity is the means to achieve faster learning by increasing the number of unstable silent synapses that may be recruited into new memories by structural plasticity and consolidation. Taken together, the model can explain the major differences of structural plasticity during development and adulthood by shifts in how metabolic constraints and learning speed are leveraged.

To simulate structural and weight plasticity we have used a simple three state Markov model of a potential synapse where state transition probabilities (with exception of 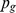) depend on a Hebbian-type consolidation signal 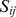 (see Fig. 2A, Eq. 4). Our plasticity model generalizes the binary Willshaw model [26], [44] and strongly simplifies realistic weight plasticity models, for example, those based on spike-timing dependent synaptic plasticity (STDP) where potentiation depends on the precise temporal order of presynaptic and postsynaptic spikes [117]–[119]. In fact, it has been discussed controversially whether STDP-type learning rules would at all be consistent with the Hebbian idea that “what fires together wires together” because, unlike the Willshaw model, simple STDP models predict decoupling of neurons firing at the same time [120]–[123]. However, we have recently shown that more realistic STDP models (including dendritic propagation delays and parameters fitted to physiological data) are generally consistent with Hebbian learning and local cell assemblies [124].

Similarly, we argue that our model is also consistent with more realistic models of structural plasticity based on homeostatic mechanisms for maintaining mean neuronal firing rates at a constant level [20], [50]. In such models, generation and elimination of synapses is induced by firing rates being below and above the homeostatic level, respectively. This is similar to our model with a homeostatic constraint for maintaining a constant anatomical connectivity 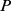 (see Section 2), because the mean firing rate of a neuron (e.g., during phases of ongoing activity [125]) will strongly correlate with the number of synapses on its dendrite (cf. [53], [126]). Thus, keeping firing rates in homeostasis is essentially equivalent to maintaining the number of synapses per neuron and, thus, 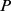, at a constant level. In our simulations, we have explicitly adjusted the generation rate 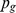 in each step in order to keep 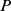 constant, but in a more realistic setting, 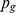 could as well be driven by factors representing each neuron's mean firing rate.

Thus, we argue that both Hebbian and homeostatic structural plasticity are necessary to optimize information storage: Hebbian structural plasticity (via 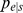) is necessary to eliminate those synapses that are not useful for storing a memory set. But homeostatic structural plasticity (via 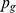) is also necessary: First, to balance the requirements of fast learning (large 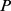) and space and energy efficiency (low 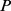). Second, homeostatic structural plasticity may also contribute to *uniformly* sample new memory representations 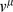 from the space of all possible activity patterns (with unit usages 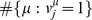 being equal for all neurons 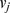), which is known to be optimal for minimizing output noise and maximizing storage capacity in multi-layer networks (see Fig. 7 in [127]; cf. [126], [128], [129]): For example, a neuron representing only a few memories will have few state-1 synapses and, correspondingly, low firing rates. This may increase 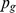 to generate new state-0 synapses, rendering this neuron more plastic and receptive for being used to represent new memories, thereby increasing state-1 synapse number and firing rates until the desired homeostatic level is reached. Some previous works have actually argued that non-Hebbian homeostatic structural plasticity could be sufficient to explain memory formation [18], [130]. Although this may hold true if cell assemblies representing different memories would be spatially separated with only little overlap, our results emphasize also the need of Hebbian-type structural plasticity with a specific elimination of unconsolidated synapses. Without Hebbian structural plasticity it seems impossible to stabilize a larger number of overlapping cell assemblies and to come close to the high memory capacity of our model [16].

By introducing the concepts of effectual connectivity 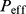 and consolidation signal 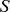, our theory remains largely independent of a specific underlying neural network model of memory. In fact, the performance of the specific model in terms of output noise 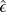 is generally a non-linear monotonic function 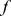 of effectual connectivity, e.g., 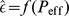, where 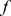 depends on the network model, network size, number of active units per memory vector, number of stored memories, and other factors. Here we have investigated Willshaw-type networks with binary synapses [16], [26], [44] because they give a simple and intuitive answer to the question which synapses are irrelevant and thus eligible for pruning. However, the efficiency of structural plasticity generalizes to learning employing graded synaptic states [53], [54], [79]. Previous approaches to memory formation by structural plasticity have also discussed that memories could be encoded in the number of synapses rather than by changing weights of individual synapses [28].

There are several lines of evidence suggesting that the binary weight model (corresponding to states 0 and 1) is already quite useful, in particular, if one would add suitable noise terms to account for distributed synaptic strength: First, experiments indicate that real synapses may have only a small number of functionally distinctive states or may even be binary [74]–[76], [131]. Second, real synapses tend to scale their strengths such that in the soma (where spikes are generated) the resulting postsynaptic potentials have a relatively constant amplitude [61]. Third, anatomical experiments have shown that the number of real synapses per connected neuron pair is relatively constant in cortical areas [59] which indicates active regulation, for example, based on spike correlations [132], [133]. Together, these findings support the hypothesis that the number of synapses per neuron pair and the strength of synapses at different dendritic locations might be co-regulated in order to keep the effect of a neuron onto a *connected* neighbor close to a desired constant magnitude. From a functional viewpoint, this perfectly makes sense at least for some functions such as memory storage (or the storage of “random” memory indices [134]) where binary synapses are optimal for storing sparse neural activity patterns [46], [53], [73].

Although our definition of effectual connectivity 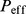 is tailored for the analysis of structural plasticity and memory storage, it shares many features with previous definitions of effective connectivity, e.g., based on “Granger causality” or “transfer entropy” used for analyzing the functional structure of brain networks from measured neural activity [135]–[137]. For example, transfer entropy 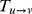 [137] is a measure of the directional information flow from one brain area 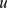 to another area 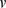. In the simplest case the transfer entropy between activities 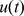 and 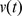 measured in two brain areas 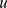 and 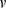 is defined as 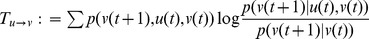 where 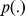 denotes the distribution of activity patterns, see Eq. 4 in [137] for details. This measure is very similar to the transinformation-based capacity measure 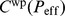 (see Eqs. 10,14) which depends monotonically on 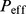 rendering effectual connectivity an equivalent measure of how well an input activity pattern 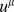 in one area can reactivate a corresponding target pattern 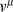 in another area. In fact, the equivalence of the two measures, 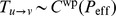, can be shown for a simplified model of neural activity propagation in brain areas [138].

Adding to previous results of storage capacity based on counting possible synaptic network configurations [28]–[30] (cf. Eq. 12), our model proves that simple memory networks of 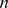 neurons with structural plasticity can indeed store *and* retrieve up to 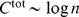 bits per synapse. By comparison, even with real-valued synapses that have an infinite number of states, Hebbian-type weight plasticity without structural plasticity achieves less than one bit per synapse [72], [73], [139], [140]. Technical adaptations of our model to applications such as information storage and pattern recognition have exhibited advantages in terms of recognition time and memory requirements compared to methods based on traditional weight plasticity [16], [53], [127].

Besides increasing storage capacity and energy efficiency of neural networks, our results suggest that structural plasticity is a key element in understanding various memory phenomena. One key prediction of the model under homeostatic maintenance of anatomical connectivity 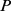 are time-dependent gradients in effectual connectivity 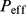, such that memories from an earlier time have higher 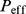 than memories from a later time. These gradients occur because consolidation of an increasing number of memories will continuously decrease the number of “migratable” (not yet consolidated) synapses and, thus, learning of new memories becomes slower and slower. We have shown that such gradients in 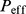 can explain both aging effects and the absence of catastrophic forgetting because learning may stop just before the number of stored memories reaches the critical capacity limit [31], [36], [99]. The same gradients in 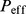 can also explain Ribot gradients in amnesic patients suffering from lesions of the medio-temporal lobe [38]–[40]. Ribot gradients can also be explained by gradients in accumulated consolidation time, assuming unlimited cortico-hippocampal consolidation [102], [104]. However, our model is unique in producing Ribot gradients even for finite consolidation times, in accordance with findings of a time-limited role of the hippocampal system in consolidation [34], [38], [39].

Last, our model is able to bridge different models, describing the spacing effect [43] on psychological [41], [42], [106] and molecular levels [141] by identifying structural synaptic plasticity as the potential cellular mechanism for spacing effects. The presence of structural plasticity in the adult brain is not only strongly supported by recent experimental evidence. As our results show, it is necessary to achieve high storage capacity and energy efficiency, and inevitably causes spacing effects. Structural plasticity is consistent with psychological theories that explained the spacing effect by encoding variability [106], [108] but attributes the increased variability for spaced rehearsal to the changing pattern of synaptic connections rather than a changing learning context. While previous models based on delayed synaptic consolidation induced by molecular signaling cascades [52], [141] may account for short-term spacing effects on the time-scale of minutes, structural plasticity can also explain long-term spacing effects on the time scale of months to years [110], [111]. As the temporal profile of optimal learning depends on parameters of structural plasticity, predictions from theories of structural plasticity will be testable by future experiments that can link memory performance (behavioral data) and structural plasticity (physiological data) in cortical areas where these memories are stored.

## Mathematical Analysis

### I Temporal Dynamics of Effectual Connectivity 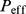


#### I.1 Relation between synapse and network states

As will be shown, effectual connectivity 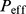 is a macroscopic network state that can be computed from the (microscopic) states of individual potential synapses. For this we first have to describe the relation between microscopic synaptic state variables 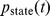 (Eq. 4) and the corresponding macroscopic connectivity variables 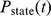. As indicated in the main text this relation is non-trivial (see text below Eq. 4), because there may be multiple actual and/or potential synapses between each neuron pair 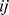, whereas connectivity of a neuron pair 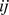 has to be defined in terms of the presence of *at least* one synapse or the absence of *all* synapses. For example, we could define neuron pair 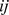 to be in state 1 if there is at least one potential synapse 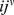 that is in state 1. Similarly, we define that 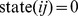 iff 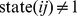 and there is at least one real synapse with 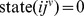. Finally, 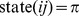 iff 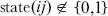 and there is at least one potential synapse with 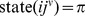.

Next we divide neuron pairs into distinct groups, where two neuron pairs are in the same group if they receive identical consolidation signals 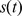. Then, in analogy to Eq. 4 we can define the (macroscopic) fractions 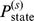 of neuron pairs 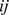 belonging to group 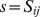 and being in a certain 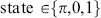,
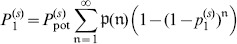
(17)

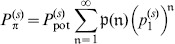
(18)

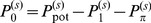
(19)where 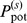 is the fraction of neuron pairs that have a potential synapse and receive consolidation signal 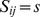 (typically 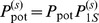 if the matrix of potential connections is independent of the stored memories), and 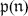 is the probability that there are exactly 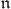 potential synapses given that there is at least one potential synapse for neuron pair 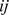. See ref. [59] for neuroanatomical estimates of 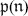 in various cortical areas.

From this we can compute the macroscopic state variables 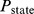 defined as the fractions of neuron pairs 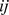 that are in a particular 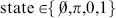 (where state 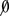 denotes neuron pairs without any potential synapses) and the various connectivity measures defined in Section 1,
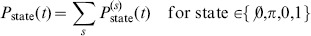
(20)

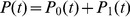
(21)

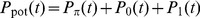
(22)

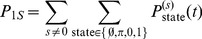
(23)

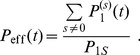
(24)


By these definitions we are in the position to do microscopic simulations of networks of potential synapses and compute the corresponding connectivity measures (e.g., as we have done for Fig. 6; see also Section 1).

While we have worked out a general theoretical framework of structural plasticity [142], the following analyses will be limited to the much simpler case where a neuron pair has at most one synapse, 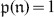. Such a setting is justified by experimental findings that there is an active regulation of the total connection strength of the synapses connecting two neurons towards a constant value (see discussion section).

#### I.2 Increase of 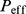 towards 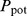


To prove Eq. 11 let us now analyze the temporal dynamics of effectual connectivity 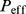 under simplified conditions. Specifically, we analyze the increase of 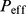 towards 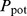 during consolidation in a *large* network with *constant* anatomical connectivity 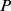 having at most a *single* potential synapse per neuron pair. For this we will assume a simple *constant* consolidation signal, i.e., ongoing rehearsal or replay with 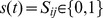 for 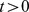. Constant 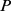 requires a *homeostatic constraint* where generation and elimination of synapses are in approximate balance,
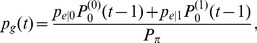
(25)where 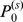 is as defined in Sect. Mathematical Analysis I.1. Furthermore, we assume 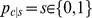 and sufficiently large neuron populations 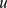 and 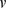 with sizes 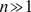 (cf. Fig. 3) such that 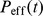 and 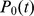 (and 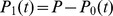) are always close to their expectations. Thus, at any point in time, there exist 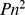 synapses distributed over 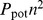 possible locations. Before learning starts, the network has already 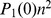 consolidated synapses (e.g., due to earlier learned memories) that are unrelated to the novel memories specified by 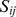. Thus, initially 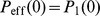 (Eq. 24). After the first learning step at 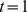 all available synapses get potentiated and consolidated, 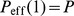. For 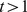 it is



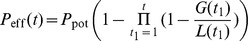
where 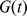 is the number of new synapses generated at time 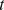 (which equals the number of eliminated synapses), 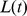 is the number of potential locations to put them, and 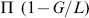 is the probability that a given potential synapse 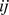 with 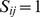 is not yet realized and consolidated until time 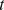. For 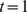 we can assume 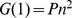 and 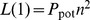. For 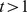 it is 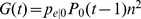 and 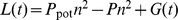, where the number of unconsolidated synapses, 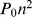, computes from



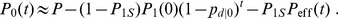



i.e., all real synapses minus initially consolidated (and not yet deconsolidated) synapses minus the newly consolidated synapses marked by 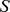. Thus, the factors in the product become 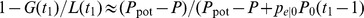. Therefore
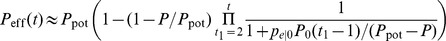



proving Eq. 11. The second approximation in Eq. 11 becomes valid if all product terms are approximately equal, i.e., if 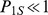 (set of novel memories is small) and 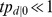 (deconsolidation during the time interval of rehearsal or replay is negligible). Note that here the increase of 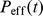 does not depend on 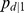 since synapses with 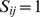 that get deconsolidated are immediately (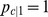) reconsolidated.

### II Evaluation of Memory Capacity

#### II.1 Asymptotic analysis for one-step retrieval

As argued in Section 6, the storage capacity of structurally plastic networks where memories are stored with effectual connectivity 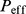 is equivalent to the capacity of a structurally static network with increased anatomical connectivity 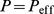 (cf. Fig. 3). Therefore the following computes the storage capacity for one-step retrieval in the Willshaw network without any structural plasticity (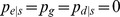, 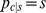; see Section 3 and Fig. 3A) where synaptic weights are given by Eq. 5.

For the following approximate asymptotic analysis we use several simplifications. First, Address and content memory patterns 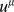, 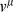 are binary random vectors of size 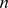 each having 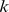 active units (i.e., 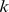 is the size of a Hebbian cell assembly representing the memory in population 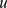 or 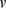). Second, the The query pattern 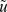 has 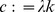 randomly chosen “correct” one-entries of an address pattern 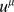 (where 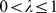) but no additional “false” one-entries (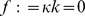). Third, as previously suggested [78], [143]–[145], we assume that each neuron 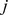 can optimize its firing threshold 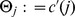 according to the number of connected active “correct” query neurons, that is, 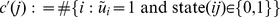.

Let us first estimate error probabilities after storing 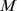 associations. We have 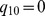 due to the assumptions of optimal threshold control and zero add noise (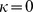). To see this note that 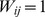 for any actual synapse 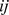 with 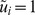 (which implies 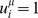 due to the zero add noise assumption) and 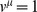. Therefore the dendritic potential 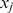 will equal 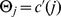 and thus 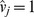 if 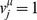. By contrast, 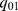 depends on the probability 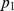 that a given synapse is potentiated (see Eqs. 4, 5). After storing 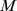 memory associations we have
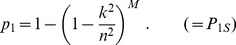
(26)


This follows from the fact that a synapse is potentiated with probability 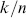 during presentation of a single memory. After presentation of all 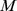 memories, the synapse will therefore still be in state 0 (unpotentiated) with probability 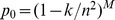. The state probability 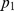 has been called “memory load” or “matrix load” in previous works [16] because, for fully connected networks, 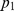 corresponds to the fraction of one-entries in the weight matrix. From Eq. 26 we obtain that a “low neuron” 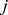 with 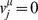 may fire with error probability
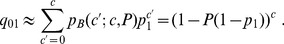
(27)where 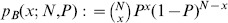 is the binomial probability. Note that 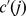 follows a binomial distribution such that 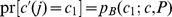. Thus, the sum in Eq. 27 averages over all possible values of 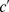 where the error probability given 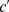 is 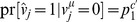. This is because an error requires that all 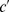 relevant synapses of neuron 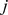 are potentiated, where the probability of one synapse being potentiated is 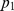. An exact analysis shows that this binomial approximation of 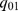 becomes exact in the limit of large networks and sufficiently small cell assemblies with 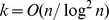 (see [129]; see also Section II.2).

Now we can compute the storage capacity by limiting output noise 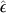 (Eq. 7) by some constant 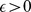. Thus, we have to solve
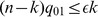
(28)


for 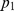 which gives the maximal matrix load 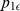 of Eq. 13 that satisfies 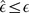. With this, solving Eq. 26 for 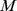 yields the pattern capacity 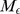 of Eq. 13. For small 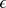 and 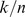 it is 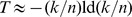 and with Eq. 10 it follows the weight capacity Eq. 14.

For networks with structural plasticity Eq. 13 is still valid but effectual connectivity will be typically larger than anatomical connectivity, 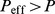. As silent synapses are functionally irrelevant and can be pruned (but see the remarks below) we can compute total storage capacity in bits per synapse from renormalizing Eq. 14. Thus, dividing the totally stored information by 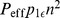 instead of 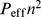 yields
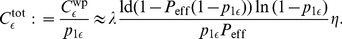
(29)


For large 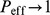 and small 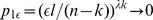 the total storage capacity per synapse diverges with network size 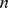,
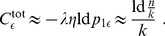
(30)


Together with Eq. 11 this proves that in networks with structural plasticity, high potential connectivity, and sufficiently small cell assembly size 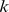, it is possible to come close to the information theoretic capacity bound (see Eq. 12).

One limitation of this analysis is the assumption of an optimal threshold control. In fact, an optimal threshold control as presumed above would actually require silent synapses in order to compute spike thresholds 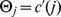 in incompletely connected *excitatory* networks with 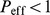 [143], [144] (so they should not be pruned). Therefore we will use the resulting expressions for 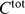 merely for approximating the storage capacity for a more conservative threshold control (see next section). Nevertheless the results are still asymptotically correct for high effectual connectivity 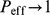 because then the optimal spike threshold 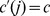 gets independent of remaining silent synapses [16]. Corresponding results hold true also for *inhibitory* network models where an optimal spike threshold control could easily be realized (including pruning of silent synapses) because it is independent of 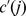 for any 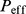 [78]. This suggests that structural plasticity could store information in inhibitory networks even more efficiently than in excitatory networks (cf. [13]).

#### II.2 Numerical evaluation for finite networks

The analysis of the previous section is asymptotically correct for large networks (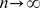), large connectivity (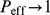), and sparse activity (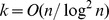) [16], [129]. It is also useful to get an overview about the qualitative effect of increasing effectual connectivity 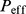 and its relation to the memory load 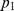. To compute storage capacity of finite networks with large activity 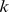 and low connectivity 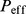 it is possible to do an exact analysis by generalizing the approach of [129]. However, as such an approach would be computationally very expensive, the following develops a Gaussian approximation of dendritic potential distributions, which can reduce reduce computation time by several orders of magnitude. For example, in some preliminary experiments we have evaluated the exact storage capacity 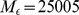 for 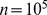, 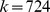, 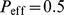 for 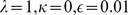 which took about 57 h on a single core of an 2.2 GHz AMD Opteron compute server. By comparison, using the Gaussian approximation developed in this section yields 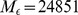, quite close to the exact value, but took only 2.5 sec computing time.

Let us first consider the Willshaw-Palm distribution 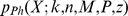 defined as the exact probability that a content neuron's dendritic potential 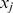 equals 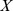 given that 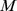 random memories are stored in a heteroassociative Willshaw-Palm network with size 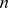, anatomical connectivity 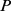, and (constant) activity 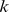 if stimulating with a random pattern (unrelated to the stored memories) with 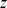 active units. From Eq. 3.22 in [129] we obtain 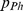 for the special case of fully connected networks (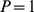),
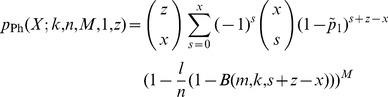
(31)where 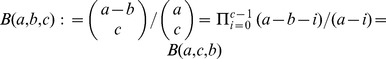. In network with general connectivity 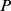 each of the 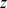 active input units is connected to neuron 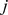 with probability 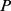. Therefore the number of connected neurons is binomially distributed and



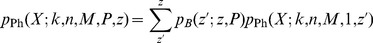
(32)We can now determine the first two moments of this distribution, The mean 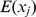 can easily be computed from the memory load Eq. 26,
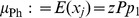
(33)and the variance



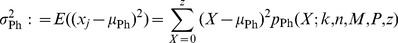
(34)

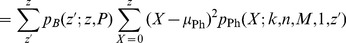
(35)

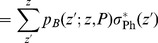
(36)


can be computed from the corresponding variance of a fully connected network which is well approximated by (see Eq. 4.25 in [129])
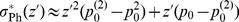
(37)


where 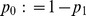 (cf. Eq. 26) and 
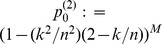
. Therefore the variance of the diluted network is well approximated by
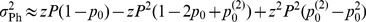
(38)


From these results we can easily compute mean values and variances of the dendritic potential distributions of high and low units. Here high units are neurons 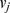 with 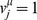, i.e., neurons that should be activated during retrieval. Similarly, low units are neurons 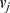 with 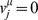. Thus, if the query pattern 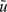 has exactly 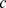 correct units from an address memory 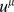 and additionally 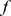 randomly chosen false units (not active in 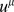) then the mean and variance of a low unit's dendritic potential will be
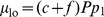
(39)

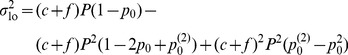
(40)and mean and variance of a high unit's dendritic potential will be



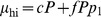
(41)

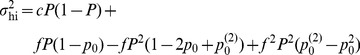
(42)


Assuming Gaussian distributions we can compute a globally optimal firing threshold 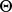 that minimizes output noise 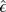 by applying some standard methods (e.g., see appendix D in [46]). Then we can determine pattern capacity 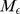 by doing a binary search to efficiently find the maximal 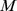 that satisfies 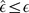. Finally, we can determine 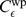 from Eq. 10 and thus also 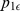 from Eq. 26 and 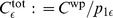. Corresponding data for 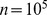 is shown in Fig. 5.
