# Correction: Structural Synaptic Plasticity Has High Memory Capacity and Can Explain Graded Amnesia, Catastrophic Forgetting, and the Spacing Effect

**DOI:** 10.1371/journal.pone.0141382

**Published:** 2015-10-19

**Authors:** The PLOS ONE Staff

There are errors in Fig 2. Please see the corrected Fig 2 here.

**Fig 2:**
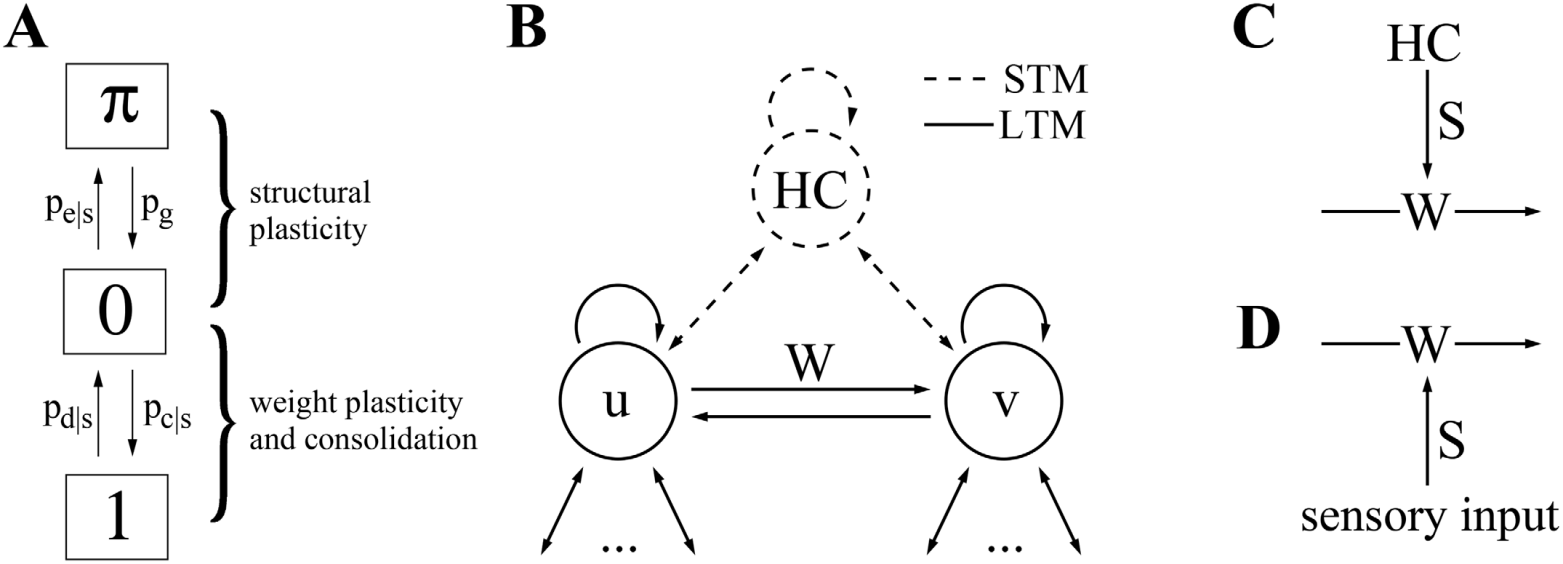
Model of structural plasticity and consolidation. **A**, State/transition model of a single potential synapse (see text for details). **B**, In the following we consider potential synapses in a network *W*, for example, connecting two cortical neuron populations *u* and *v*. Memories correspond to associations between activity patterns *u* ^*μ*^ and *v* ^*μ*^. We will specifically analyze how well noisy activity patterns ${\tilde{u}}^{\mu }$ can reactivate the corresponding memories *v* ^*μ*^ in order to estimate storage capacity. **C, D**: LTM storage (solid) by structural plasticity requires repetitive reactivation of activity patterns in cortical populations *u* and *v* to provide an appropriate consolidation signal *S* to the synapses. This may happen by repeated bottom-up stimulation (**D**) or, for episodic memories, by top-down replay (**C**) from a HC-type STM buffer (dashed). LTM = long-term memory; STM = short-term memory; HC = hippocampus.

There are errors in Fig 3. Please see the corrected Fig 3 here.

**Fig 3:**
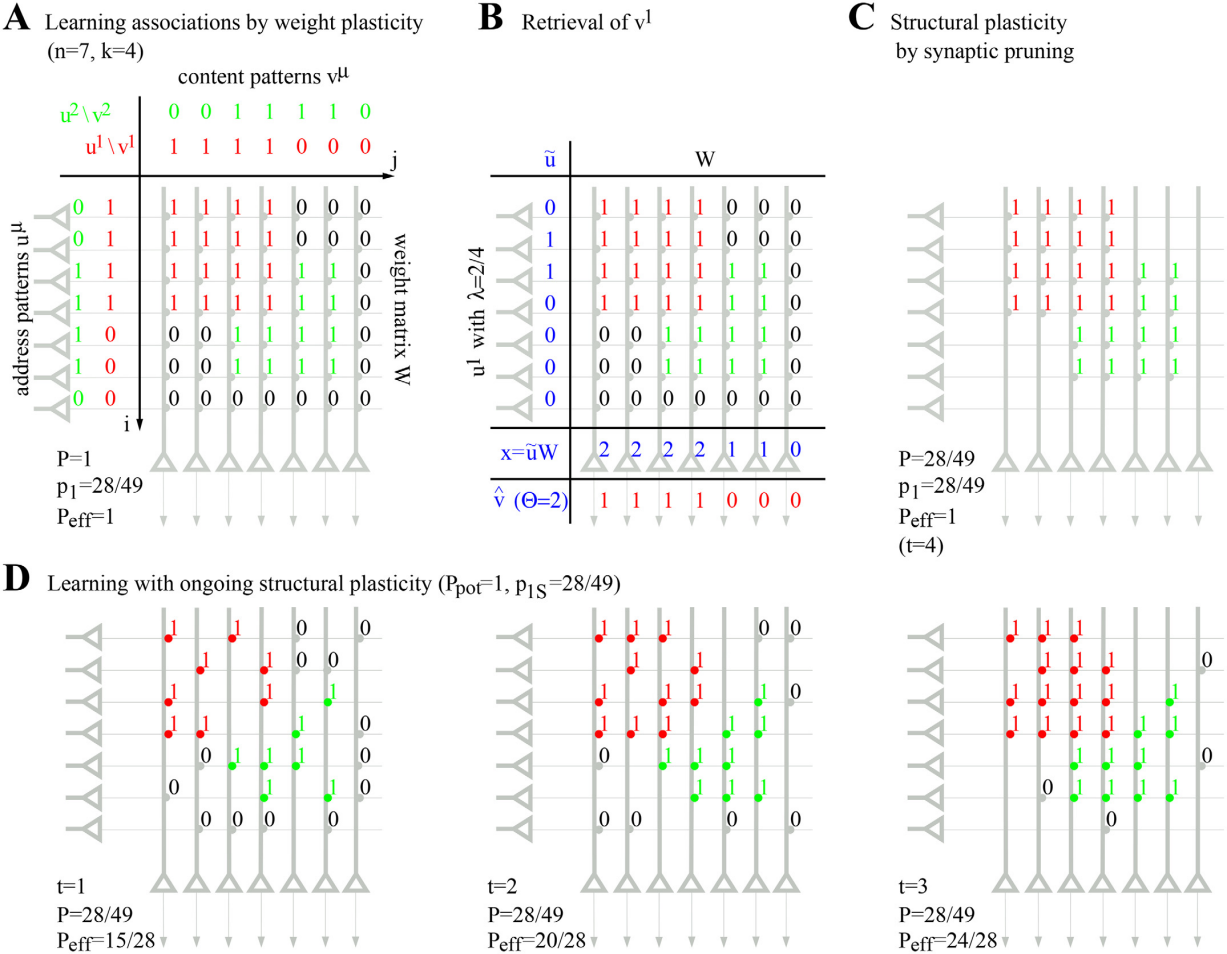
Learning in Willshaw-type associative networks. **A**, Memory storage by Hebbian weight plasticity (Eq. 5) in a fully connected network (*P* = 1). Address patterns *u* ^*μ*^ are associated to content patterns *v* ^*μ*^ where *μ* = 1,…,*M* (here *M* = 2). Each memory is represented by a binary activity vector of length *n* = 7 having *k* = 4 active units (which define the corresponding cell assembly). **B**, One-step retrieval of the first memory from a noisy query pattern $\tilde{u}\approx u^{1}$ having two of the four active units in *u* ^*1*^ (*λ* = 0.5). Here $\tilde{u}\approx u^{1}$ can perfectly reactivate the corresponding memory pattern in population *v* ($\hat{v}=v^{1}$) applying a firing threshold $\Theta =\sum_{i}{\tilde{u}}_{i}=2$ on dendritic potentials $x_{j}=\sum_{i=1}^{m}W_{ij}{\tilde{u}}_{i}$. **C**, As a simple form of structural plasticity, silent synapses can be pruned *after* learning. The resulting network has only 28 (instead of 49) synapses corresponding to a lower anatomical connectivity *P* ≈ 0.57, whereas the effectual connectivity is still *P*_eff_ = 1. Thus, pruning does not change network function, but increases stored information per synapse. **D**, Ongoing structural plasticity can similarly increase storage capacity during more realistic learning in networks with low anatomical connectivity (here *P* = 28/49 ≈ 0.57). During each time step *t* = 1, 2, 3, 4, Hebbian weight plasticity potentiates and consolidates synapses *ij* with non-zero consolidation signal *S*_*ij*_ > 0 (which equals *W*_*ij*_ of panel A), whereas the remaining silent synapses are eliminated and replaced by new synapses at random locations. Note that the resulting network at *t* = 4 is the same as in panel C.

There are errors in Fig 6. Please see the corrected Fig 6 here.

**Fig 6:**
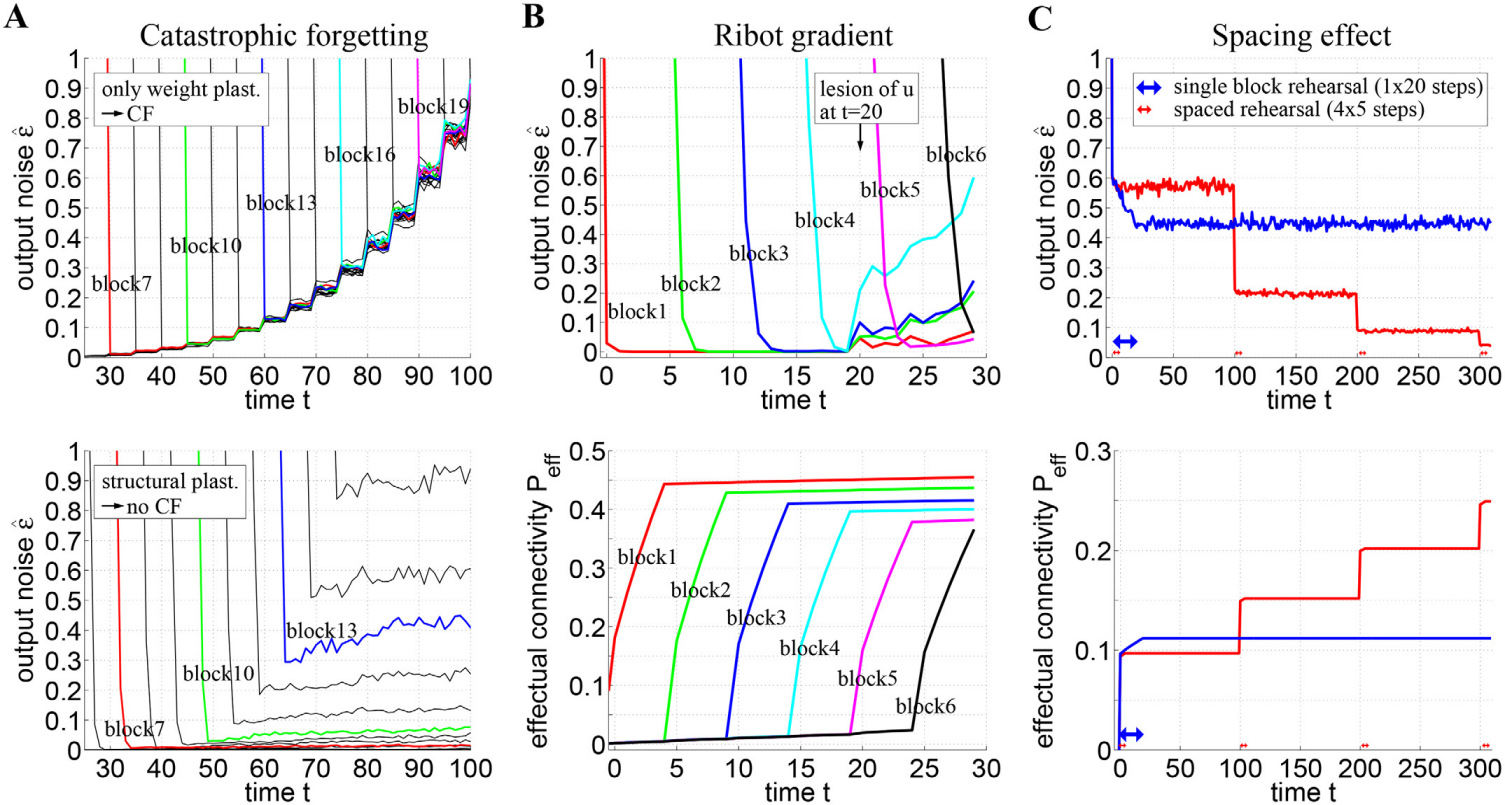
Simulation of catastrophic forgetting, Ribot gradients, and the spacing effect. **A**, Networks without structural plasticity suffer from catastrophic forgetting (top), but networks with structural plasticity do not (bottom). Plots show output noise $\hat{\epsilon }$ over time *t* simulating networks of size *n* = 1000 and activity *k* = 50 storing 25 memory blocks one after the other (only the interesting part between storage of blocks 6 and 21 are visible). Each curve (with a distinct color) corresponds to $\hat{\epsilon }$ for noisy test patterns of a particular memory block with *c* = 45 correct and *f* = 5 false active units. The steep descent of each curve corresponds to the time when the Hippocampus started to replay the corresponding memory block for 5 time steps. **B**, Networks employing structural plasticity show Ribot gradients after a cortical lesion (top) due to gradients in effectual connectivity (bottom). The lesion was simulated by deactivating half of the neurons in population *u* at time *t* = 20. **C**, Networks employing structural plasticity reproduce the spacing effect of learning. In the first simulation (blue) novel memories were rehearsed once for 20 time steps (blue arrow at *t* = 0−19). In a second simulation (red) the same total rehearsal time was “spaced'' or distributed to four brief intervals of five steps each (red arrows at *t* = 0−4, *t* = 100−104, *t* = 200−204, and *t* = 300−304). Here the network achieves a higher effectual connectivity *P*_*eff*_ (bottom) and less retrieval noise *ϵ* (top). See Sections 2, 3 and Table 1 for further details and simulation parameters.

The publisher apologizes for these errors.
